# Snowflake Iron Oxide Architectures: Synthesis and Electrochemical Applications

**DOI:** 10.3390/molecules29204859

**Published:** 2024-10-14

**Authors:** Anna Kusior, Olga Waś, Zuzanna Liczberska, Julia Łacic, Piotr Jeleń

**Affiliations:** 1 Faculty of Materials Science and Ceramics, AGH University of Krakow, al. Mickiewicza 30, 30-059 Kraków, Poland; zuzanna.liczberska@gmail.com (Z.L.);; 2 Faculty of Electrical Engineering, Automatics, Computer Science and Biomedical Engineering, AGH University of Krakow, al. Mickiewicza 30, 30-059 Kraków, Poland; olgawas@student.agh.edu.pl; 3 Faculty of Energy and Fuels, AGH University of Krakow, al. Mickiewicza 30, 30-059 Kraków, Poland; julia.lacic65@gmail.com

**Keywords:** snowflakes, hematite, electrochemical sensors, dopamine, shape-engineering, Fe_2_O_3_

## Abstract

The synthesis and characterization of iron oxide nanostructures, specifically snowflake architecture, are investigated for their potential applications in electrochemical sensing systems. A Raman spectroscopy analysis reveals phase diversity in the synthesized powders. The pH of the synthesis affects the formation of the hematite (α-Fe_2_O_3_) and goethite (α-FeOOH). Scanning electron microscopy (SEM) images confirm the distinct morphologies of the particles, which are selectively obtained through recrystallization during the elongated reaction time. An electrochemical analysis demonstrates the differing behaviors of the particles, with synthesis pH affecting the electrochemical activity and surface area differently for each shape. Cyclic voltammetry measurements reveal reversible dopamine detection processes, with snowflake iron oxide showing lower detection limits than a mixture of snowflakes and cube-like particles. This research contributes to understanding the relationship between iron oxide nanomaterials’ structural, morphological, and electrochemical properties. It offers practical insights into their potential applications in sensor technology, particularly dopamine detection, with implications for biomedical and environmental monitoring.

## 1. Introduction

Over 85 years ago, Wagner and Hauffe discovered that adsorbed molecules on the surfaces of semiconductors may affect their properties [1]. An interruption of the crystal lattice periodicity (size decreasing, geometrical changes) affects the increase in free dandling bonds, the incomplete coordination number, and thus the surface rearrangement and reactivity. New prospects in material design enable the effective interaction and charge transfer between various biomolecules and electrode surfaces. Moreover, electrocatalytic activity determines the reaction efficiency and may improve detection sensitivity.

So far, a typical biosensor is considered a biological molecule receptor-targeted recognition of enzymes [2], antibodies [3], or aptamers [4]. The use of this type of compound has many advantages, such as biocompatibility, high binding affinity, high catalytic efficiency, and reasonable specificity. Appropriately designed nanomaterials can mimic the behavior of enzymatic materials [5]. Signal reproducibility, long lifetimes, and stability set them apart. Moreover, they exhibit distinct physicochemical properties, such as a high electroactive surface area and facile charge carrier transfer between the target molecule and the electrode surface, which can quickly improve the sensing performance.

The key to designing materials is to direct their synthesis properly. Using surfactants, the presence of foreign ions, or a suitable reaction environment results in nanostructure deformation, and changes in surface chemistry state may occur [6,7,8]. Parameters such as the diffusion coefficient, electron transfer rate, charge transfer, or electrochemical active surface area depend on the interaction between the electrolyte/analyte and the receptor. The receptor materials’ characteristics may actively contribute to the interaction with the molecules by promoting adsorption. However, near the neutral pH, the observed reaction may only contribute to the surface state of the materials and may not be affected by the medium.

However, the stability of biomolecules, e.g., dopamine (DA) and their oxidation form, is also determined by environmental conditions [9,10]. Dopamine, as a hormone, plays an important modulatory role in the central nervous system. They are responsible for critical aspects such as motor functions and nervous, hormonal, and cardiovascular systems [11]. Alteration in normal dopaminergic neurotransmission underlies multiple neurological diseases, including schizophrenia, attention deficit hyperactivity disorder (ADHD), Huntington’s disease, and Parkinson’s disease [12]. The modulation of dopamine-regulated signaling pathways is also crucial in the addictive actions of most drug abusers. While many techniques, such as liquid chromatography [13], fluorescence [14,15], and surface plasma resonance [16], are available for dopamine detection, most of the attention has been focused on finding inexpensive and easy-to-use detectors with high sensitivity and fast response times. Therefore, electrochemical methods, among other possibilities, have been extensively developed [17,18,19].

So far, it has been confirmed that pH is the rate-limiting process in DA cyclization [20,21]. However, the electrochemical reaction rate is significantly affected by the nature of the electrode surface.

One solution to increase the reaction rate involves a catechol moiety (a benzene ring with two hydroxyl groups), which can form strong interactions with receptor surfaces through hydrogen bonding and coordination interactions. This correlation is widely used in iron oxide-based materials for biomedical applications [22,23]. The affinity between the iron ions and hydroxyl groups enhances selective binding and sensing. Moreover, they can facilitate electron transfer reactions, which can affect the oxidation of dopamine to its quinone form [24,25,26,27]. Moreover, by controlling the synthesis parameters, the preferable size and geometry of the particles can be obtained [28]. Enhancing the surface-to-volume ratio improves the interaction between dopamine molecules and the sensor’s surface, enabling the detection of very low DA concentrations. A crucial parameter is the stability of the iron oxides under a range of physiological conditions.

This article focuses on synthesizing and characterizing iron oxides as nanostructures with a snowflake-like architecture for electrochemical applications, particularly dopamine sensors. This study showed that synthesis parameters, such as pH and reaction time, have a crucial impact on the morphology and electrochemical properties of the materials, which directly translates into their effectiveness in dopamine detection. The novelty of this work is the investigation of the effect of particle shape on the dopamine detection process, which may have important implications for developing sensors for biomedical applications. This research was undertaken to improve the performance and sensitivity of electrochemical detectors based on the iron oxide nanomaterials’ (receptor) interaction with dopamine based on the coordination interaction.

## 2. Results and Discussion

### 2.1. Iron Oxide Powder Characterization

The reaction condition usually drives the product’s morphology. Hydrothermal treatment of the K_3_[Fe(CN)_6_] solution at various OH^−^ concentrations should affect its dissociation and further material crystallization. Herein, two series of iron oxide-based materials were synthesized: (i) series S, without pH modification, where used compounds affected the solution (pH = 8.5), and (ii) series P with an increased pH of up to 12. For both series, materials were synthesized at 24, 48, and 72 h.

**Figure 1:**
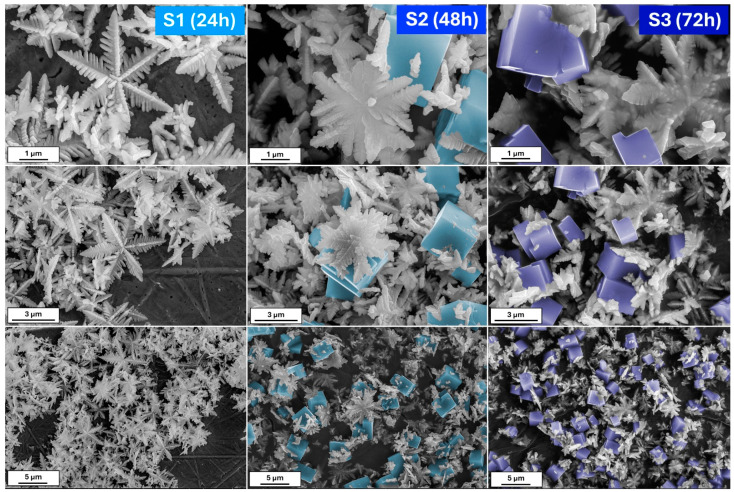
The SEM images of the obtained iron oxide nanostructures (S series) after 24, 48, and 72 h from the solution with 8.5 pH.

Figure 1 shows the scanning electron microscopy (SEM) images of iron oxide-based architectures obtained by hydrothermal treatment of a K_3_[Fe(CN)_6_] solution at pH 8.5. After 24 h of reaction, the powder consists of hexagonal snowflake particles. The increase in the synthesis time to 48 h results in the formation of 3D stars; however, cubic forms are also visible. The presence of the damaged particles allows us to conclude that due to the Oswald ripening process, some of the particles dissolve and recrystallize [29]. On the other hand, the elongated reaction affects the mass transfer. Therefore, it can be assumed that plane hexagonal snowflake iron oxides transform into 3D structures, first as stars and then as cubes. When the synthesis reaches 72 h, the cubic form dominates.

**Figure 2:**
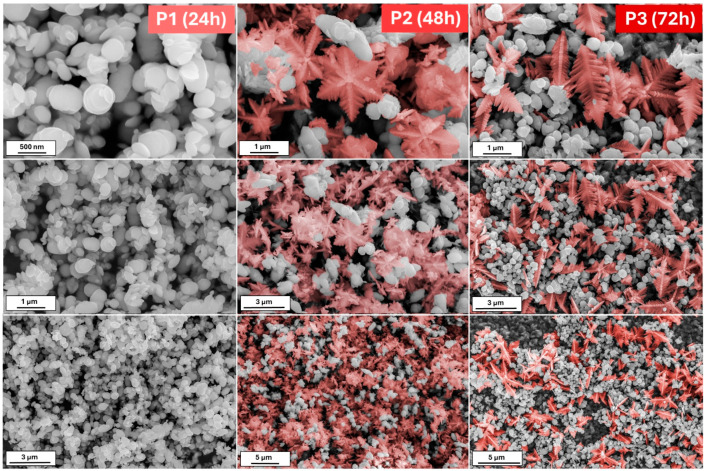
The SEM images of the obtained iron oxide nanostructures (P series) after 24, 48, and 72 h from the solution with 12 pH.

A small change in the process of obtaining the material, such as an increase in the reaction’s pH, allows the observation of something completely different. Figure 2 shows the morphologies of the powder obtained after 24, 48, and 72 h from the K_3_[Fe(CN)_6_] solution of pH = 12. In contrast to the previous series, herein, after the 24 h process, the nanoplates of iron oxides are visible. With a prolonged reaction time, thick hexagonal snowflakes appear. Their size is smaller than in the series S. The large amount of nanoplates remains. However, the snowflakes are damaged when the reaction is extended (72 h). Except for the nanoplates, the single branches may be distinguished.

**Figure 3:**
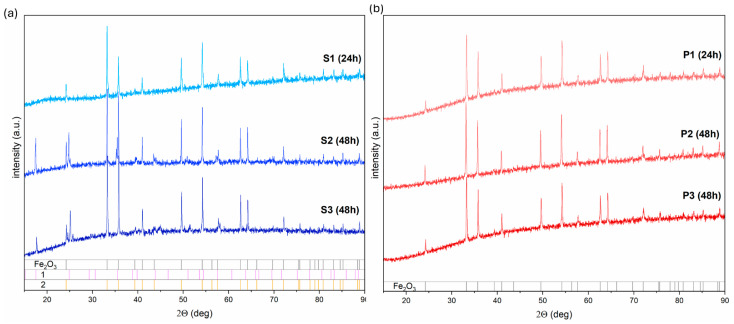
The XRD analysis of the iron oxide-based material obtained (**a**) at pH = 8.5 and (**b**) at pH = 12. Caption 1 is assigned to the tetrairon(III) hexacyanoferrate(II) 9.3-hydrate 4.7-(dideuriohydrate), and 2 to tetrairon(III) tris(hexacyanoferrate(II)) tetradecahydrate.

An X-ray diffraction analysis (XRD) can provide more information about the materials’ behavior and synthesis (Figure 3a,b).

The 24 h process in series S affects the formation of the pure hematite structure (PDF# 01-085-0987). No other additional peaks due to the possible impurity phases were noticed. Highly stable in room temperature, ions [Fe(CN)_6_]^3−^ at a higher temperature treatment for a long time undergo dissociation processes into Fe^3+^ ions, which can, upon further hydrolysis, get converted into hydroxy oxides and then hematite [30,31]. However, the presence of the tetrairon(III) hexacyanoferrate(II) 9.3-hydrate 4.7-(dideuriohydrate) and tetrairon(III) tris(hexacyanoferrate(II)) tetradecahydrate (PDF #98-002-3103, PDF #98-000-1272) suggest that the number of the OH^−^ is insufficient to complete the microstructure transformation from hematite snowflakes to hematite cubic particles for elongated synthesis (48 and 72 h).

The addition of the OH^−^ to the K_3_[Fe(CN)_6_] solution (pH = 12) increases the dissociation rate of [Fe(CN)_6_]^3−^ and facilitates crystallization in α-Fe_2_O_3_ (Figure 3b):

(1) $$[Fe(CN{)}_{6}{]}^{3-}↔Fe^{3+}\to FeOOH/Fe(OH{)}_{3}\to \alpha \text{-}Fe_{2}O_{3}$$

**Figure 4:**
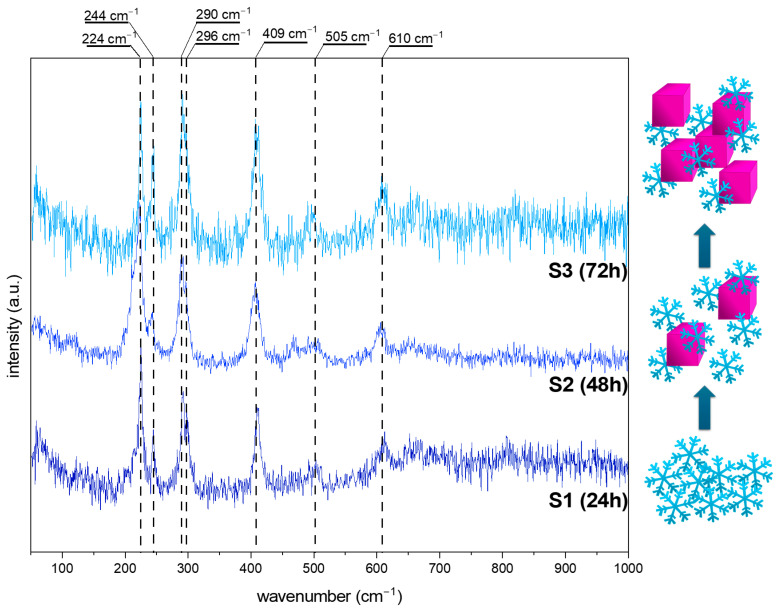
The Raman spectra of the obtained iron oxide nanostructures (S series) after 24, 48, and 72 h from the solution with 8.5 pH. The scheme of the obtained structures at various synthesis times.

**Figure 5:**
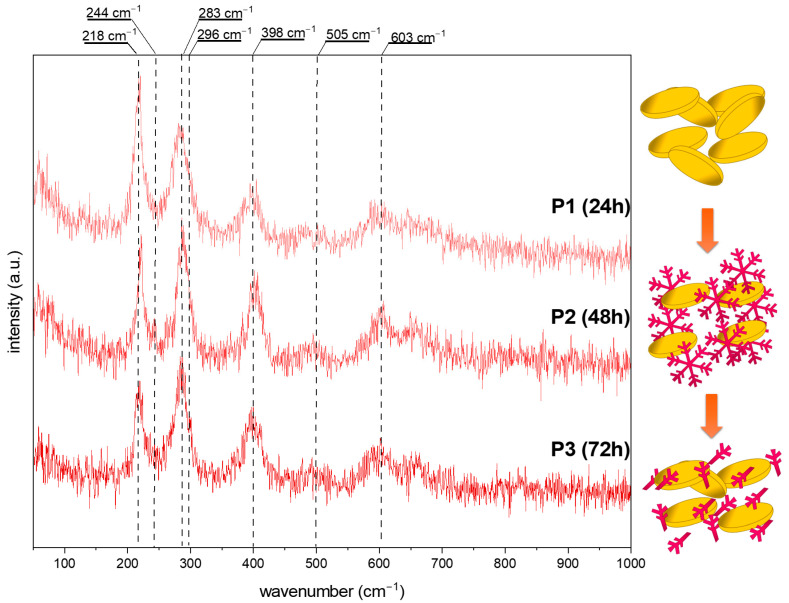
The Raman spectra of the obtained iron oxide nanostructures (P series) after 24, 48, and 72 h from the solution with 12 pH. The scheme of the obtained structures at various synthesis times.

The intermediate phase of hydroxyoxides may be confirmed by using more sensitive techniques, such as Raman spectroscopy, especially when the amount is under the XRD’s detection limit. Additionally, because tetrairon (III) hexacyanoferrate(II) 9.3-hydrate 4.7-(dideuriohydrate) and tetrairon (III) tris(hexacyanoferrate(II)) tetra decahydrate are ionic compounds, their presence in the structure will be neglected. Figure 4 and Figure 5 show the Raman spectra of the obtained iron oxide architectures.

Figure 4 shows data obtained for the series S. For all spectra, vibration bands characteristic of hematite (α-Fe_2_O_3_), at around 224, 290, 409, 505, and 610 cm^−1^, are visible [32,33]. Additional bands at 244 and 296 cm^−1^ may be assigned to the goethite form (α-FeOOH) [32,34,35]. The relation between the modes at 224 and 244 cm^−1^ while the elongated reaction time is worth noting. After 24 h of synthesis, the ratio of 1.5:1 suggests the dominance of hematite. The particle transformation from snowflakes to cubes requires a mass transfer, thus resulting in a three times higher signal from FeOOH in the Raman spectra. The 72 h process allows recrystallization, and the amount of α-Fe_2_O_3_ increases. All data are summarized in Table 1.

An almost pure α-Fe_2_O_3_ composition is presented for the series P (Figure 5). The modes related to the goethite show a too low signal or overlap with the characteristic vibrational bands of hematite. However, the reaction time effect is also visible. With a prolonged synthesis, the band’s intensity at about 218 cm^−1^ decreases, which may be related to the structure/particle decomposition/deformation (Table 1).

Comparing the above results, it may be concluded as follows: (i) The molar ratio of K_3_[Fe(CN)_6_] and OH^−^ affects the crystallization of the iron oxide architectures and phase composition. In contrast, the molar ratio K_3_[Fe(CN)_6_]:OH^−^ 1:7 affects the formation of the snowflake shapes (24 h)—the ratio of 1:12 results in the production of nanoplates. The hydroxyl ions affect the material’s growth mechanism, according to Zhang et al. [36], the OH^−^ is also responsible for surface modification. Higher concentrations of OH^−^ prevent the O^2−^ surfaces from further reaction. Therefore, flat and smooth crystal facets are obtained (nanoplates). The growth mechanism is related to the dissociation rate of [Fe(CN)_6_]^3−^ and further reaction with hydroxyl groups. (ii) The reaction time controls and tunes the morphologies of the iron oxide-based particles.

### 2.2. Electrochemical Characterization of Obtained Materials

**Figure 6:**
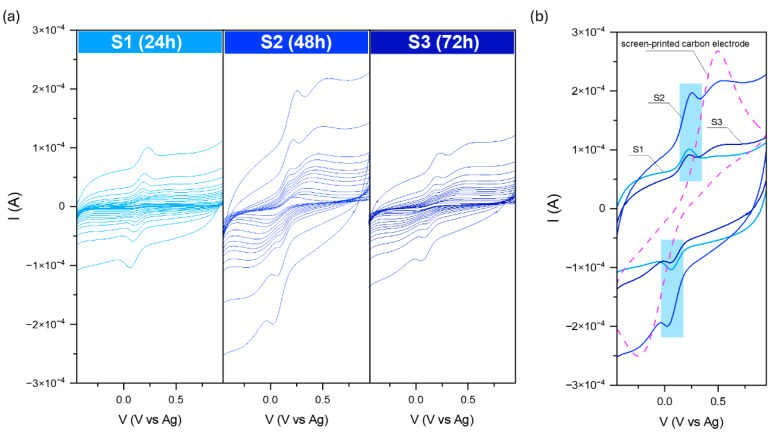
Current–voltage dependence for the modified carbon electrodes (**a**) in the 0.1 M KCl + 3 mM [Fe(CN)_6_]^3−/4−^, and comparison with the SPE (**b**). Data were recorded at various scan rates from 10 to 2000 mVs^−1^.

**Figure 7:**
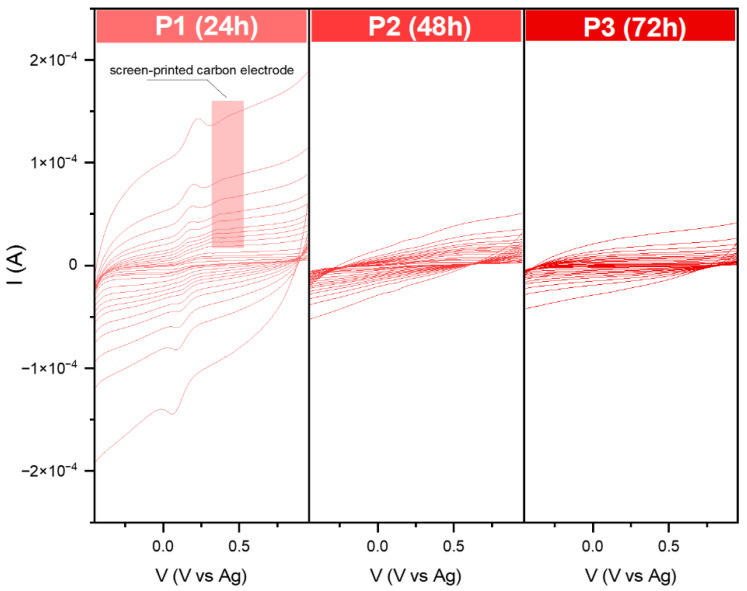
Current–voltage dependence for the modified carbon electrodes in the 0.1 M KCl + 3 mM [Fe(CN)_6_]^3−/4−^ solution. Data were recorded at various scan rates from 10 to 2000 mVs^−1^.

**Figure 8:**
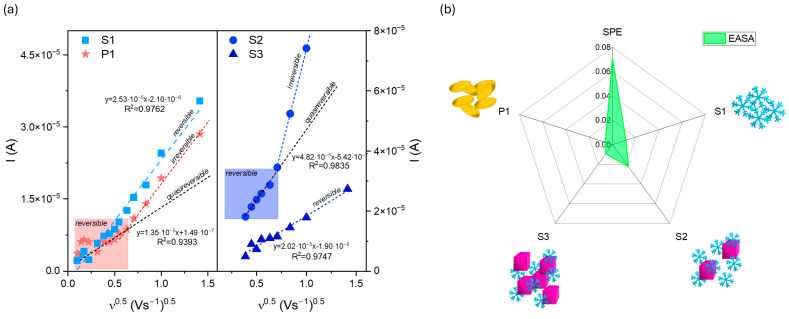
(**a**) The plot of the anodic peak current as a function of the square root of the scan rate ν^0.5^ for modified electrodes with (**b**) electrochemically active surface area, EASA, compared to the SPE electrode.

Powders from both series were obtained and deposited on the screen-printed electrodes (SPEs) using the drop-casting technique. They were then analyzed for electrochemical activity in the redox couple 0.1 M KCl + 3 mM [Fe(CN)_6_]^3−/4−^ solution. The results are summarized in Figure 6, Figure 7 and Figure 8. Ferricyanide/ferrocyanide undergoes oxidation and reduction repeatedly without significant changes in the system’s behavior or the electrochemical reaction mechanism. When an electric potential is applied to this solution, [Fe(CN)_6_]^3−^ is reduced to [Fe(CN)_6_]^4−^, and similarly, [Fe(CN)_6_]^4−^ is oxidized back to [Fe(CN)_6_]^3−^.

The current–voltage dependence for the S series-modified electrodes is presented in Figure 6. For all samples, characteristic peaks at the anodic (Ia) and cathodic (Ic) curves related to the [Fe(CN)_6_]^3−/4−^ redox reaction are visible; however, the highest signal was recorded for S2. Moreover, additional broad peaks were visible for the samples received for the elongated reaction time (48 and 72 h). They originated from a very thin receptor layer at the screen-printed electrodes and its imperfect covering of the SPE substrate. The comparison of the recorded signal for pure SPE and the modified electrodes allows for the assignment of each peak for the right processes. Further analysis of the received current at the maximum (ratio Ia/Ic) may suggest that all observed behavior is reversible or quasi-reversible. The calculated ratios were 1.19, 0.86, and 1.06 for S1, S2, and S3, respectively. Moreover, under these conditions, the reversibility of the reaction is this ideal separation value of about 50 mV. For all recorded data, the separation was from 5 to 35 mV.

Surprisingly, for the series P, the oxidation–reduction peak of the [Fe(CN)_6_]^3−/4−^ can be observed only for P1. As in the case of the previously modified screen-printed electrodes, the peak from the target is also visible here. The recorded data for P2 and P3 are similar to each other. The receptor layer seemed thin, so no additional peaks from SPE could be distinguished. On the other hand, according to the received banana shape, it could be the effect of the high capacitive (background current), which could have covered the voltametric signal.

The analysis of the peak current ratio for P1 shows that Ia/Ic is approximately 0.95. However, the plots of the anodic peak current as a function of the square root rate ν^0.5^ (Figure 8a) reveal the complexity of the as-prepared electrodes’ behaviors.

The Randles–Ševčik equation was used to determine the electrochemically active surface area (EASA):

I = 2.69 × 10^5^ × EASA × D^0.5^ × n^2/3^ × C_0_ × ν^0.5^ (2)

where D is the diffusion coefficient of the [Fe(CN)_6_]^3−/4−^ redox species (7.2 × 10^−6^ cm^2^s^−1^), n is the number of the electrons involved in the reaction, herein 1, C_0_ refers to 3 mM of the concentration of the redox species, and ν represents the scan rate (Vs.^−1^). The obtained data are summarized in Figure 8b. Implementing the obtained powders at the surface of the carbon substrate results in a decrease in EASA. The SPE electrochemical active surface area is about 0.071 cm^2^, while for S1, S2, S3, and P1, its size equals 0.012, 0.022, 0.009, and 0.006 cm^2^, respectively. The results are unsatisfactory despite the shape complexity and various exposed crystal facets. On the one hand, it proves that the receptor modified the SPE surface. Conversely, the mesoporous structure formed from connected snowflake particles seems inactive or presents a repulsive interaction between the redox couple and material.

While the [Fe(CN)_6_]^3−/4−^ environment is considered a benchmark redox system for electrochemical characterization (e.g., EASA, diffusion coefficient) as a redox probe and neutral pH condition, phosphate-buffered saline (PBS) is close to the biological conditions required. Therefore, the sensing properties were evaluated in a 0.1 M PBS solution with dopamine (DA) molecules.

**Figure 9:**
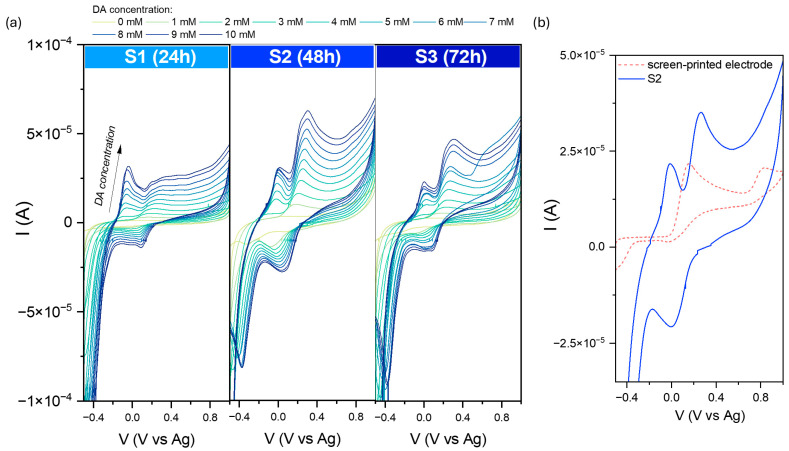
(**a**) Comparison of cyclic voltammograms in various dopamine concentrations (from 1 to 10 mM solution in PBS environment) for modified screen-printed electrodes by the obtained iron oxide powders. (**b**) The recorded data at 5 mM DA for the pure SPE and S2-modified electrode. Data were recorded at 50 mVs^−1^.

Figure 9 shows the cyclic voltammograms recorded after subsequent DA additions in the 0–10 mM concentration range. As observed for all samples, the dopamine detection process is reversible. However, an additional strong peak at higher potential values is visible. Its presence can be explained by an inhomogeneous distribution of the receptor layer using the drop-casting technique. The location of the peak and its intensity suggest that it comes directly from the SPE itself, which is also sensitive to dopamine molecules. Moreover, the process of DA detection of SPEs is irreversible, which can be confirmed by the absence of the related peak and the cathodic curve. These data are related to observed electrochemical behavior in the [Fe(CN)_6_]^3−/4−^ environment.

Basic parameters characterizing the sensor activity, such as the limit of detection (LOD), and quantification (LOQ) were calculated by using the following relations:

LOD = 3.3 SD × a^−1^ (3)

**Figure 10:**
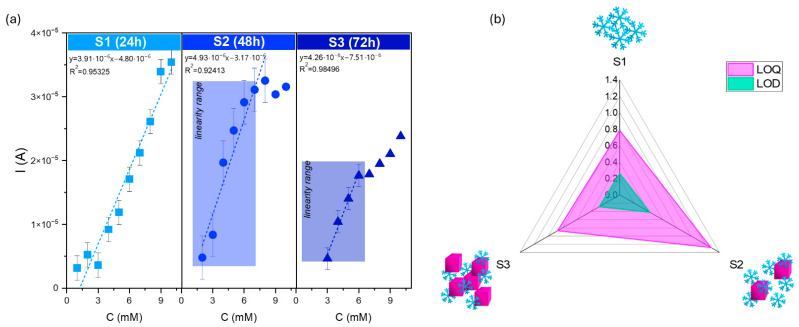
(**a**) Calibration curves for series S modified screen-printed electrodes in the presence of various concentrations of dopamine (from 1 to 10 mM solution) with (**b**) LOD and LOQ parameters of the SPE-modified electrodes. The square, circle, and triangle symbols correspond to the dopamine changes for S1, S2, and S3 samples.

LOQ = 10 SD × a^−1^ (4)

where SD is a standard deviation obtained from the slope measured current vs. dopamine concentration (Figure 10a), and a is the slope of the linear regression plot.

Figure 10b summarizes the parameters characterizing the electrochemical performance. The snowflake particles’ lower LOD and LOQ values were determined (S1). According to this research’s aim, the results confirm that the specificity of this architecture affects the presence of a large number of active sites, which play crucial roles in molecule detection.

**Figure 11:**
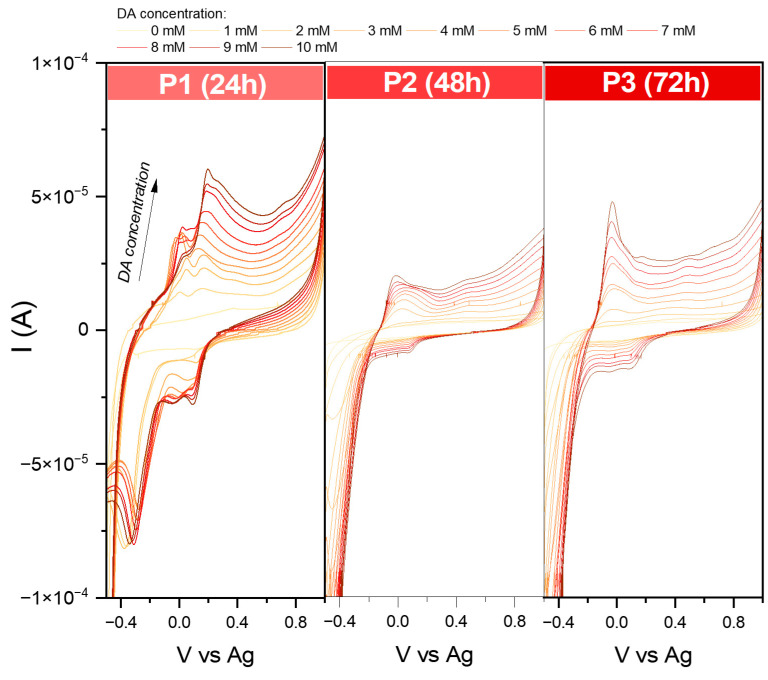
Comparison of cyclic voltammograms in various dopamine concentrations (from 1 to 10 mM solution in PBS environment) for modified screen-printed electrodes by the obtained iron oxide powders. Data were recorded at 50 mVs^−1^.

While for series S, it was possible to define the LOD and LOQ for the essential characterization of sensing properties, several problems were encountered for the series P (Figure 11). First, in sample P1, during the measurements, the receptor layer was partially dissolved; therefore, the recorded curves are full of interferences. Even though during the analysis of the EASA, no redox reaction was observed for the P2 and P3, the modified screen-printed electrodes showed activity towards dopamine. Still, the received Ia and Ic suggested the irreversibility mechanism. Moreover, in the case of P2, the shape of the obtained curve indicated that the detection process is based on electron transfer, followed by a chemical reaction [37,38]. A similar effect may be observed for P3. The recorded signal summarizes overlapping two or more peaks [39]. The complexity of the mechanism detection at this stage is not easy to define. This problem needs to be further investigated.

The lack of stability of the receptor layer completely rules out the usefulness of the P series as dopamine sensors. Nevertheless, a careful analysis of the electrochemical behavior may make it possible to explain the reason for this phenomenon.

The situation is different for the S series. Consideration should be given to (i) the geometry of the powder itself, and (ii) the influence of impurities in the form of the tetrairon (III) hexacyanoferrate(II) 9.3-hydrate 4.7-(dideuriohydrate) and tetrairon (III) tris(hexacyanoferrate(II)) tetra decahydrate, which are ionic compounds. First, ideal snowflake materials, although they pose poor EASA, the complexity of the structures and exposure to various facts affect their activity towards dopamine. Moreover, the hematite structure may affect the interaction between receptor surfaces through hydrogen bonding and coordination interactions with dopamine molecules. The elongated synthesis without the additional presence of OH^−^ groups results in unreacted materials, which, as can be assumed, block this repulsive bonding, and the activity of the sensors drops. Due to this fact, the shape effect cannot be analyzed as trustworthy.

However, comparing the obtained data with the literature (Table 2) indicates that the snowflakes’ complex structure has the potential to be a dopamine sensor. The LOD and LOQ values compared to widely used electrodes based on carbon materials are similar. Moreover, their electrochemical behavior is better than that of other metal oxides.

## 3. Experimental Section

### 3.1. Synthesis

Nanostructures were synthesized by a hydrothermal reaction of the potassium ferricyanide, K_3_[Fe(CN)_6_] with different pH values and time at 180 °C. First, K_3_[Fe(CN)_6_] (POCH, Gliwice, Poland) was dissolved in distilled water. Initially, the pH was stabilized at 8.5 (S series). The adjustment to 12 was made using a basic NaOH (POCH, Gliwice, Poland) aqueous solution (P series). After stirring for 15 min, the mixture was transferred to the Teflon-lined stainless-steel autoclave with a capacity of 250 mL and kept at 180 °C for 24, 48, and 72 h. After the reaction, the obtained powder was centrifugated, washed with water and ethanol (50%/50% *v*/*v*), and dried under vacuum at 40 °C for 24 h. Synthesis parameters and sample assays are summarized in Table 3.

### 3.2. Material Characterization

The sample morphology was analyzed using scanning electron microscopy (SEM, ThermoFisher Scientific Scios 2, Waltham, MA, USA). The structure’s size was determined using the ImageJ program (version 1.54g).

The crystal structure of the obtained materials was investigated using an X’Pert MPD diffractometer (Malvern Panalytical Ltd., Malvern, Worcestershire, UK). The system worked in the Bragg–Brentano geometry. Phase identification was performed using X’Pert HighScore Plus software (version 3.0.4) and the Powder Diffraction File (PDF-2).

The Horiba Scientific LabRam HR (Horiba Scientific, Tokyo, Japan) examined the powders’ structural properties. It was equipped with a 532 nm laser and grating 1800, 50× objective. Each measurement took 20 s, and the laser power was set to 0.3 mW to prevent sample degradation and/or phase transition.

Modified carbon screen-printed electrodes were prepared using the following procedure: 8 mg of the powder was sonicated in a 1 mL solution of isopropyl alcohol, distilled water, and Nafion in a 2:7:1 volume ratio. Afterward, 2 µL of the mixture was drop-casted on a clean electrode (Metrohm DropSens, Oviedo (Austrias) Spain) and left to dry in air at room temperature. As formed, the receptor was further analyzed by the cyclic voltammetry (CV) technique to define their electrochemical properties.

The electrochemical surface area, EASA, was determined by measurements performed in a 0.1 M KCl + 3 mM [Fe(CN)_6_]^3−/4−^ electrolyte at different scan rates (ν, 10–2000 mVs^−1^). Dopamine detection tests were performed in a 0.1 M PBS (phosphate-buffered saline) solution in the potential range from −0.5 to 1.0 V at a constant scan rate of 50 mVs^−1^. All electrochemical measurements were conducted using an electrochemical analyzer Interface 1010 TM Potentiostat/Galvanostat/ZRA (Gamry Instruments, Warminster, PA, USA).

## 4. Conclusions

This study highlights the significant influence of the synthesis parameters, such as reaction time and pH, on the morphology and phase composition of iron oxide-based architectures and their subsequent electrochemical properties. The morphological evolution observed in the SEM images confirms that increasing the reaction time leads to a transition from hexagonal snowflake particles to 3D structures, such as stars and cubes, with distinct differences noted between samples synthesized at different pH levels. The XRD data reveal the role of OH^−^ groups in forming the pure hematite phase. The Raman spectroscopy results further corroborate these morphological changes, revealing a dynamic interplay between hematite (α-Fe_2_O_3_) and goethite (α-FeOOH) phases, depending on the synthesis conditions. An electrochemical characterization of the synthesized materials demonstrated variable performance in redox systems and dopamine sensing, with the series S showing a more defined and stable electrochemical response than the series P. The challenges encountered with series P, such as receptor layer instability and complex detection mechanisms, underscore the need for further optimization and investigation. However, the promising results obtained for series S, particularly the lower limit of detection (LOD) and limit of quantification (LOQ) for dopamine sensing, suggest that the specific architecture of the snowflake particles enhances the sensor’s activity due to the presence of a large number of active sites. This study demonstrates the crucial role of synthesis conditions in determining iron oxide-based materials’ structural and functional properties. These findings offer valuable insights into designing and optimizing nanostructured materials for electrochemical applications, particularly sensor development.

## Figures and Tables

**Table 1:** Raman shift and intensity for the selected bands of iron oxide-based architectures.

Series	Sample	Shape	Hematite		Goethite	
			Raman Shift [cm^−1^]	Intensity [a.u]	Raman Shift [cm^−1^]	Intensity [a.u]
**series S** **(pH = 8.5)**	**S1**	snowflake	225	88.86	244	55.59
	**S2**	snowflake +cubes	223	33.16	243	99.32
	**S3**	snowflake +cubes	224	59.52	244	47.21
**series P** **(pH = 12)**	**P1**	nanoplates	220	92.43		
	**P2**	snowflakes +nanoplates	220	64.19	242	38.84
	**P3**	nanoplates +branches	218	22.10		

**Table 2:** Comparison between screen-printed electrode-based sensors towards dopamine detection.

Electrode Material	LOD	LOQ	Linear Range	Ref. ^1^
AuNps@MoS_2_ ^2^	0.21 µM	-	3–20 µM	[40]
activated SPE	0.067 µM	-	0.2–30 µM	[41]
hand-made SPE	1.25 μM	4.17 μM	5–100 μM	[42]
Au	0.22 μM	-	2–100 μM	[43]
alkali-activated graphitized carbon	0.25 μM	-	1–150 μM 150–1000 μM	[44]
molecularly imprinted polymers	0.8 μM	2.0 μM	0.8–45 μM	[45]
SPCE-Fe_3_O_4_/SPEEK ^3^	7.1 μM	-	5–50 μM	[46]
Co_3_O_4_:Fe_2_O_3_	0.24 μM	-	-	[46]
Fe_3_O_4_	19.3 μM		10–61 μM	[47]
Fe_2_O_3_ snowflakes	0.26 μM	0.78 μM	1–10 μM	this work
Fe_2_O_3_ snowflakes + cubes	0.42 μM	1.28 μM	1–7 μM	this work
Fe_2_O_3_ snowflakes + cubes	0.29 μM	0.87 μM	1–6 μM	this work

^1^ Ref.—reference; ^2^ Nps—nanoparticles; ^3^ sulfonated polyether ether ketone-Iron (III) oxide modified screen-printed carbon electrode.

**Table 3:** Synthesis parameters and sample assays.

Synthesis Parameter		S Series			P Series		
		S1	S2	S3	P1	P2	P3
chemical reagent	K_3_[Fe(CN)_6_]	1.06 g			0.59 g		
	DIW ^1^	160 mL			120 mL		
pH		8.5			12.0		
reaction time (h)		24	45	72	24	48	72
temperature (°C)		180			180		

^1^ DIW—distilled water.

## Data Availability

The dataset is available upon request from the authors.
